# Comparing Neighborhood Indices of Socioeconomic Status, Segregation, and Healthcare Access for Predicting Late-Stage Breast Cancer

**DOI:** 10.1007/s11524-026-01072-4

**Published:** 2026-04-10

**Authors:** Matthew R. Dunn, Srijon Mukhopadhyay, Scarlett L. Gomez, Lauren E. McCullough, Terry Hyslop, Marc A. Emerson, Melissa A. Troester

**Affiliations:** 1 Department of Epidemiology, University of North Carolina, 253 Rosenau Hall, CB #7435, 135 Dauer Drive, Chapel Hill, NC 27599 USA; 2 Lineberger Comprehensive Cancer Center, University of North Carolina, Chapel Hill, NC USA; 3 Department of Epidemiology and Biostatistics, University of California, San Francisco, CA USA; 4 Department of Epidemiology, Rollins School of Public Health, Emory University, Atlanta, GA USA; 5 Thomas Jefferson University, Sidney Kimmel Cancer Center, Philadelphia, PA USA; 6 Department of Pathology and Laboratory Medicine, University of North Carolina, Chapel Hill, NC USA

**Keywords:** Health equity, Area-level, Census-tract-level, Deprivation, Access

## Abstract

Composite indices of neighborhood-level social environment are frequently used in cancer research, but relationships among these indices have been seldom studied, especially in race-stratified analyses. We considered four census tract-level social indices: Neighborhood Deprivation Index (NDI; continuous, quartiles, and clusters); Yost (continuous, quartiles, and clusters); racialized-income Index of Concentration at the Extremes (ICE; continuous, quartiles, and clusters); and a care access metric (categorical). These were linked to the Carolina Breast Cancer Study, a cohort of 2998 adults (50% Black, 50% aged < 50 years) with invasive breast cancer, diagnosed 2008–2013 in North Carolina, USA. Age-adjusted associations of each index with late-stage breast cancer (stage III–IV) were assessed using generalized linear models and relative frequency differences (RFDs) and 95% confidence intervals (CIs). There were strong correlations (> 0.79 for pairwise comparisons) between the continuous NDI, Yost, and ICE, but pairwise agreement was moderate (55% to 70%) among indices as quartiles. Care access had low concordance with the other three indices. Among Black participants, late-stage breast cancer was associated with all indices, including the most disadvantaged quartile (vs least) of NDI (RFD = 7.0%, 95% CI: 1.6, 12.4), Yost (RFD = 8.8%, 95% CI: 3.3, 14.3), and ICE (RFD = 7.6%, 95% CI: 2.2, 13.0) and was strongly associated with care access (RFD = 9.4%, 95% CI: 3.8, 15.0). These associations were not significant among non-Black participants. Overall, multiple neighborhood-level social indices, including one based on healthcare data, are associated with late-stage breast cancer among Black participants, suggesting multiple dimensions of vulnerability to structural barriers.

**Supplementary Information:**

The online version contains supplementary material available at 10.1007/s11524-026-01072-4.

## Introduction

Multidimensional indices of neighborhood-level social environment show stronger associations with health outcomes compared to single variables (e.g., poverty), likely because they encompass multiple socio-economic indicators [1–3]. These indices are an important tool for disparities research and have illuminated community-level differences in cancer outcomes in the United States (USA), including breast cancer mortality excesses in areas with greater deprivation [4–10]. There are many such indices available to researchers, each of which comprises different component variables and thus may capture different attributes of neighborhoods [11]. It is important to understand whether nuanced differences affect predictive value and whether different indices offer distinct value.

Prior studies have found modest-to-high correlations between indices of neighborhood socioeconomic status (nSES), but these have conducted comparisons at a geographic unit level (such as census tract and census block group) rather than person-level [12–15]. Similarly, many evaluation studies of area-level indices have only used area-level outcomes (ecologic design) [16–18]. To our knowledge, only one study has compared the performance of multiple indices—including sensitivity and relation to individual-level outcomes—in a population of adults with breast cancer [10]. Moreover, these indices measure correlated but conceptually different constructs, adding nuance to their predictive performance: for example, the Index of Concentration at the Extremes (ICE) measures the spatial concentration of groups defined by racial or economic privilege, while the Neighborhood Deprivation Index (NDI) and Yost index measure the interactions of indicators of deprivation in neighborhoods. Further research is needed to understand these patterns when applied in different racial groups and in comparison to additional neighborhood attributes. We were particularly interested in how such measures of general neighborhood deprivation would perform compared to more specific measures of disadvantage, including discrimination (given documented racial disparities in breast cancer outcomes and our study’s high representation of Black women) and access to care, given the important role of routine screening and primary care in achieving early detection and diagnosis [19].

We compared four distinct census tract-level indices in a population-based cohort of breast cancer patients in North Carolina, USA. The NDI [20] and Yost [21] measure nSES, and the racialized-income Index of Concentration at the Extremes (ICE) measures inequality and segregation [22]. These three indices were considered in their continuous, quartile, and cluster form. The fourth index (“care access”) is a latent class measure of neighborhood healthcare access that has not been compared to other published indices [23]. The objectives of this study were (1) to assess census tract- and patient-level concordance between indices; (2) to estimate the association of each index with frequency of late- (III–IV) versus early- (I–II) stage breast cancer (a key prognostic factor for breast cancer survival); and (3) to compare index performance between Black and non-Black women.

## Methods

### Study Population

The Carolina Breast Cancer Study (CBCS) Phase III is a population-based cohort of 2,998 women with breast cancer in North Carolina, USA. The study population is about 15% rural and encompasses 44 counties (out of 100 state-wide) in eastern and central North Carolina. Patients with a first primary breast cancer diagnosis between 2008 and 2013 were eligible for inclusion and were identified from the state cancer registry. The CBCS used a randomized recruitment with oversampling for Black and younger women, resulting in a cohort where half of the study population is Black and half are aged less than 50 years at diagnosis. Race was measured by participant self-report, consistent with the interpretation of race as a social construct rather than a measure of genetic ancestry [24]. Participants were grouped as Black (*n* = 1495) or non-Black (*n* = 1503); the latter group included 82 women who did not identify as White: American Indian (*N* = 12), Asian (*N* = 33), or another race not listed on the survey (*N* = 37). Detailed methodology for the CBCS has been described previously [25]. Participants were linked to census tract-level indices based on their home residence at diagnosis. All study protocols were approved by the Institutional Review Board of the University of North Carolina at Chapel Hill (Reference ID 413140).

### Community-Level Indices

We compared four census-tract level indices that were selected to capture nSES, segregation, and access to healthcare (Table S1). The Neighborhood Deprivation Index (NDI) and Index of Concentration at the Extremes (ICE) were computed using the “ndi” R package[26] based on the 2010 American Community Survey (ACS) 5-year estimates. The ICE was available as five different indices; we chose the ICE index which captures extreme concentrations of both income and racial/ethnic composition (white non-Hispanic and Black race). The Yost index based on 2014–2018 ACS data was downloaded from the University at Albany School of Public Health (https://www.albany.edu/~fboscoe/yost/). We selected this time range for the Yost as it was closest to participant time at diagnosis among available datasets. The NDI, ICE, and Yost were downloaded as continuous variables, and we generated quartiles for each index such that the 4th quartile reflects the top 25% most disadvantaged census tracts nationally.

The fourth index was developed using latent class methods [23] “Care access” measures healthcare access by area-level affordability (e.g., median income and rates of health insurance) and geographic accessibility (e.g., proximity to hospitals and doctors). For this index, census tracts were classified as high or low for affordability and accessibility, resulting in four categories.

### Outcome Assessment

The outcome of interest was AJCC tumor stage at diagnosis, binarized as early (I–II) or late stage (III–IV), obtained from participant medical records. We selected this outcome to compare SES indices for multiple reasons. First, stage is an important prognostic factor given that later stage breast cancer diagnoses have increased risk of mortality and recurrence [27] Also, diagnostic stage can reflect access to preventive care services and has been shown to correlate with detection and/or diagnostic timeliness [28]. In prior work from the CBCS, we found patients lacking mammography screening and regular primary care were more likely to have late-stage disease [29].

### Statistical Analysis

Correlations between the continuous NDI, Yost, and ICE were assessed at the census tract and participant level, overall and by race, using Pearson correlation coefficients. We calculated percent agreements for exact quartile match and for most disadvantaged quartile match—quartile 4 (Q4) vs quartile 1–3 (Q1–3). Mosaic plots were generated using the “ggmosaic” R package to graphically compare participant-level agreement between the quartile and latent class indices.

We used generalized linear models (identity link) to compute relative frequency differences (RFDs) with 95% confidence intervals (CIs) to assess age-adjusted associations of each index with late-stage breast cancer. We also performed a sensitivity analysis with adjustment for individual poverty status defined by the 2010 federal poverty level, based on self-reported income and household size. Models were assessed for the overall study population and stratified for Black and non-Black patients. The most advantaged quartile was selected as the referent group for the NDI, Yost, and ICE. For care access, categories were ordered based on the frequency of poor care quality outcomes in previous work [23]. We designated the high affordability, high accessibility areas as the referent group (most advantaged) followed by high affordability, low accessibility; low affordability, high accessibility; and low affordability, low accessibility (most disadvantaged). We also compared the commonly used quartile grouping method for the NDI, Yost, and ICE to a clustering grouping method that may better identify natural breaks in score distributions on a patient level. We used k-means clustering to separately classify participants into four groups (for ease of comparison to the 4-category latent class index) based on their NDI, Yost, and ICE scores. We generated dot plots to compare groupings by this clustering method vs. the national quartiles, and then assessed associations of the cluster-based groupings with late stage. Analyses were performed using RStudio version 12.0 (R Foundation for Statistical Computing, Vienna, Austria).

## Results

### The NDI, Yost, and ICE

A larger share of Black compared to non-Black participants were classified as NDI Q4 (38% vs 12%), Yost Q4 (44% vs 16%), and ICE Q4 (49% vs 14%) (Table S2). Correlations between the NDI, Yost, and ICE are shown in Table 1. There was a strong correlation (0.79 or higher) between each pair of indices at both the census tract- and patient level. Among all three indices, census tract agreement was 41.0% for exact quartile and 77.5% for most disadvantaged quartile (vs. all others). At the individual level, exact agreement was slightly higher among Black participants compared to non-Black (55% vs 49%), while agreement for the most disadvantaged quartile was slightly lower among Black participants (79% vs 86%). In pairwise comparisons, agreement was highest between the NDI and Yost (correlation > 0.9) and slightly lower between the ICE and NDI/Yost.

**Table 1:** Census tract- and participant-level agreement among NDI, Yost, and ICE indices

Measures being compared	Correlation coefficient (continuous score)	% agreement (exact quartiles)	% agreement (most disadvantaged quartile)
	**Census tract-level comparison **(*N* = 2195)		
All 3	n/a	41.0	77.5
NDI and ICE	−0.79	60.6	86.7
ICE and Yost	0.81	54.1	82.4
NDI and Yost	−0.92	60.7	85.9
	**Participant−level comparisons **(all participants, *N* = 2997)		
All 3	n/a	52.2	82.7
NDI and ICE	−0.84	65.6	88.4
ICE and Yost	0.86	67.5	88.6
NDI and Yost	−0.94	67.0	88.4
	(Black participants, *N* = 1495)		
All 3	n/a	55.3	79.3
NDI and ICE	−0.81	65.6	85.9
ICE and Yost	0.84	71.9	86.8
NDI and Yost	−0.93	68.5	85.6
	(Non−Black participants, *N* = 1502)		
All 3	n/a	49.3	86.1
NDI and ICE	−0.85	65.6	90.9
ICE and Yost	0.86	63.2	90.3
NDI and Yost	−0.93	65.5	90.9

**Fig. 1:**
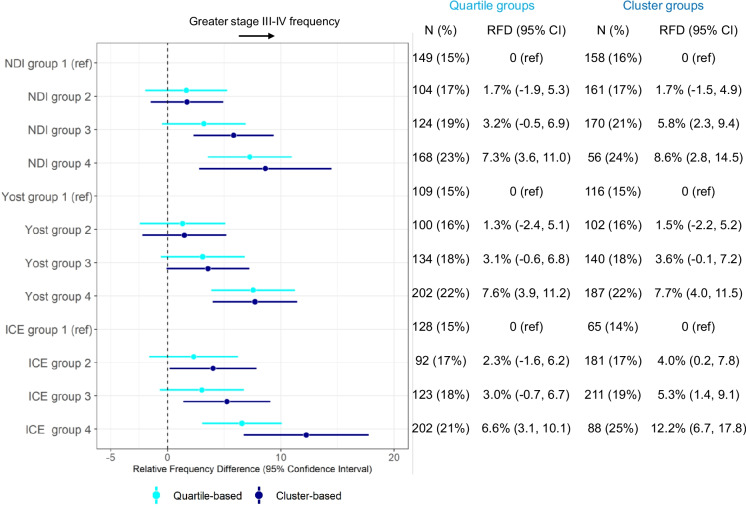
Late-stage associations with neighborhood measures grouped as quartile vs. clustering. Linear-risk regression models were used to compute age-adjusted relative frequency differences (RFDs) with 95% confidence intervals (CIs). Each estimate shows the frequency of late-stage (III–IV) relative to early-stage (I–II) cancer among the corresponding group compared to the reference (ref) group. Results are shown for the NDI, Yost, and ICE measures, categorized by national quartile cut points (light blue) and k-means clustering (dark blue). For each measure, group 4 represents the quartile or cluster with participants experiencing the most disadvantage

Each index was associated with late-stage breast cancer (Fig. 1). Late-stage breast cancer was more frequent among participants living in most disadvantaged quartile of NDI (RFD = 7.3%, 95% CI: 3.6, 11.0); Yost (RFD = 7.6%, 95% CI: 3.9, 11.2); and ICE (RFD = 6.6%, 95% CI: 3.1, 10.1) compared to the most advantaged quartile. Associations were attenuated, but still statistically significant, in sensitivity analyses adjusted for individual poverty (Table S3). We also evaluated these associations with the NDI, Yost, and ICE grouped using participant-level k-means clustering. Classification was similar between grouping methods for the Yost whereas classifications based using the NDI and ICE differed (Fig S1). The quartile-based classification resulted in a wider range of scores within upper and lower quartiles in both NDI and ICE, and therefore a narrower range of scores defined the middle quartiles. In contrast, the range of scores for cluster-based groups was more uniform. As a result, cluster-based groupings resulted in stronger associations (Fig. 1). For example, the association of ICE with late stage was stronger in magnitude using cluster-based groupings rather than quartiles. The RFD (95% CI) was 12.2% (6.7, 17.8) for the most disadvantaged cluster compared to 6.6% (3.1, 10.1) for the most disadvantaged quartile.

### Comparisons to Care Access

**Fig. 2:**
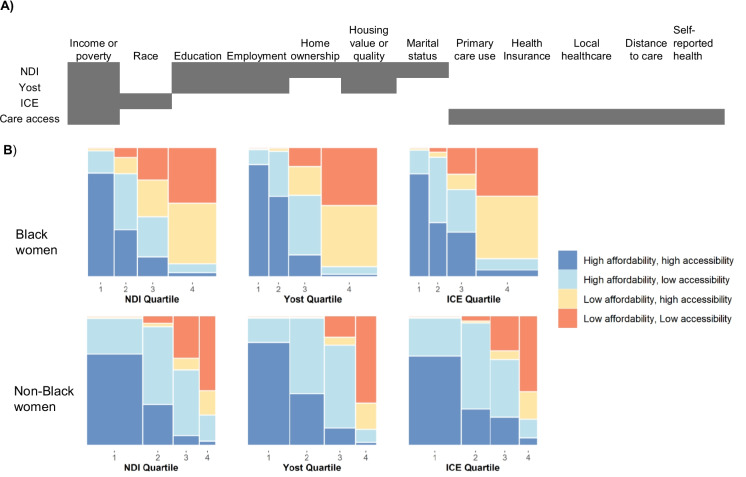
Participant classification by NDI, Yost, and ICE quartiles compared to the care access latent class. **A** A comparison of the thematic composition of the NDI, Yost, ICE, and care access measures. **B **Participant classification by NDI, Yost, and ICE compared to care access is represented by mosaic plots. Columns represent quartiles for each index where the 1st quartile signifies highest advantage and the 4th quartile signifies highest disadvantage. Each color indicates a different class of affordability/accessibility

We recently published a health care access neighborhood index [23] that leverages distinct variables compared to NDI, Yost, and ICE. This “care access” index focuses on healthcare affordability and geographic accessibility that are not captured by the other metrics (Fig. 2A). Race-stratified participant classifications are shown in Fig. 2B. Black participants in low accessibility, low affordability areas made up 44% of those classified as NDI Q4, 46% of Yost Q4, and 36% of ICE Q4. When both low affordability groups are combined, these proportions rise to 92%, 94%, and 87%, respectively. It is also noteworthy that the healthcare access index offers a distinct perspective on neighborhood disadvantage compared to the other three indices. For example, Black participants with low affordability made up 54% of NDI Q3 and 13% of NDI Q2. For non-Black participants, the two low affordability groups made up 78% of NDI Q4, 89% of Yost Q4, and 80% of ICE Q4. A lower proportion of non-Black participants with low affordability were classified in the Q1–3 of the NDI, Yost, and ICE (e.g., non-Black participants were 41% of NDI Q3 and 7% of NDI Q2).

**Fig. 3:**
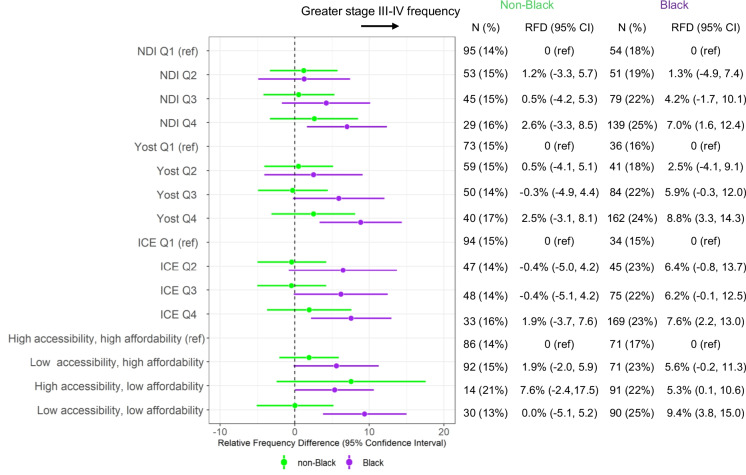
Race-stratified frequency of late-stage breast cancer and neighborhood indices linear-risk regression models were used to compute age-adjusted relative frequency differences (RFDs) with 95% confidence intervals (CIs). Each estimate shows the frequency of late-stage (III–IV) relative to early-stage (I–II) cancer among Carolina Breast Cancer Study participants, comparing the corresponding group to the indicated reference (ref) group. Results are shown for Black participants (purple) and non-Black participants (green). The NDI, Yost, and ICE measures were categorized based on the national quartile cut points, where the 4th quartile represents the most disadvantaged census tracts. Care access latent classes of accessibility/affordability were originally defined as a categorical variable

Race-stratified models for the association between neighborhood indices and stage at diagnosis are presented in Fig. 3. For each index, a significant association with late stage was observed for Black participants but was attenuated and non-significant among non-Black participants. For example, the Yost Q4 versus Q1 late-stage RFD (95% CI) was 8.8% (3.3, 14.3) for Black participants and 2.5% (−3.1, 8.1) for non-Black participants. Similar patterns were observed for the NDI and ICE. Overall, the Yost, NDI, and ICE quartiles performed similarly; however, even the second ICE Q2 showed a borderline association with late stage: (RFD = 6.4%, 95% CI: −0.8, 13.7) whereas the NDI Q2 (RFD = 1.2%, 95% CI: −3.3, 5.7) and Yost Q2 (RFD = 0.5%, 95% CI: −4.1, 5.1) did not. The highest magnitude of association (among all 4 indices) was observed for the low accessibility, low affordability latent class group (RFD = 9.4%, 95% CI: 3.8, 15.0 compared to high accessibility, high affordability).

The only high-magnitude association with late stage among non-Black participants was observed for the high accessibility, low affordability group (RFD = 7.6%, 95% CI: –2.4, 17.5), although this difference was imprecise and not statistically significant. We also note that for each index, Black participants in the most advantaged areas had a similar frequency of late stage compared to non-Black participants in the least advantaged areas. For example, late-stage prevalence was 18% for Black participants in NDI Q1 compared to 16% for non-Black participants in NDI Q4.

## Discussion

We compared three commonly used indices of neighborhood social environment (NDI, Yost, and racialized-income ICE) and observed strong correlation at the census tract- and participant-level among their continuous scores. These indices have been compared at the census tract level previously; however, the observation that these scores had somewhat lower correlation and agreement in Black participants of CBCS reflects greater heterogeneity in neighborhood factors in this population. Previous comparisons of the NDI, Yost, and ADI at the census tract (and not individual level) have shown that these metrics tend to be highly concordant [10]. Other investigations have shown modest correlation between the ADI and Social Vulnerability Index [12], and between the ADI and Yost Index [13]. However, when we compared these indices as quantiles (which are most commonly used in linking data to outcomes), there was only moderate agreement (50% to 70%) and patient-level agreement among the NDI, Yost, and ICE was only 52%. This suggests barriers to comparing results across indices, especially when small differences in neighborhood scores around categorical cut points can result in different classifications. There was, however, greater agreement among patients in the most disadvantaged quartile (82.7% three-way agreement among the NDI, Yost, and ICE for Q4), which was lower among Black vs. non-Black patients (79% vs. 86%).

The NDI, Yost, and ICE each showed distinct associations with late-stage breast cancer by race. All three indices were significantly associated with late stage only among Black women. This finding may reflect the importance of neighborhood status for Black women, a group which faces greater barriers to preventive healthcare (particularly breast cancer screening and primary care) that may act as a safeguard to neighborhood-level exposures. Furthermore, the indices considered in our analysis may correlate with additional unmeasured neighborhood effects (e.g., cumulative stress of structural racism) that particularly impact Black women. Different patterns may be observed with other outcomes and settings, including a recent study in Georgia which found the NDI was only associated with breast cancer mortality among White women [30]. However, that study also found that Black women in the most advantaged communities (per NDI) had worse outcomes compared to non-Black women in the most disadvantaged neighborhoods. While index performance has been seldom compared across racial groups, there is evidence that neighborhood indices offer different value in distinct subpopulations. Multiple prior studies have found differences in sensitivity of area-level measures when applied in different geographic areas [31–33], and performance in urban compared to rural areas is a particular concern [34, 35]. One possible limitation of neighborhood indices based on economic factors generally is that they do not adequately capture associations with healthcare related factors.

Our latent class measure of healthcare access included healthcare-specific information such as health insurance, care utilization (including mammography use), and proximity and density of healthcare resources – making it distinct from the NDI, Yost, and ICE. Associations with late stage were similar for care access compared to the other indices, but among Black participants, the low affordability, low accessibility group showed the largest magnitude of association (RFD = 9.4%, 95% CI: 3.8, 15.0) of all four indices. Also, the high accessibility, low affordability latent class was the only group to show high magnitude (albeit imprecise) associations with late-stage among non-Black participants (RFD = 7.6%, 95% CI: −2.4, 17.5). Care access identified a larger proportion of participants (36% of all participants in the two low affordability groups, and 51% of Black and 20% of white women) compared to NDI, Yost, and ICE which found only 25–30% of participants in their lowest quartile. While these differences do not imply superiority of one index over others, they emphasize that neighborhood disadvantage is complex and manifests in multiple characteristics. Consideration of differences among them could improve interpretation. Our results suggest that neighborhood healthcare access identifies areas where patients face barriers to timely cancer diagnosis and treatment.

Our results also invite consideration of additional ways to operationalize continuous neighborhood indices (vs. census tract quantile form, typically quartiles or quintiles). Application of these indices in categorical form has the advantage of producing more interpretable effect measures (e.g., risk differences and risk ratios) and it is possible to standardize cut points to facilitate comparisons across studies. However, categorization assumes homogeneity within categories, an assumption not well-supported in our data [36]. The highest and especially the lowest quartiles of NDI and ICE comprise a wider range of scores compared to the middle quartiles. Another advantage of a quantile-based approach is bolstered statistical power with equal numbers in each group. However, since cut points are defined based on the national census tract distribution, the groups were unequal once applied in the CBCS. For example, 31.5% of patients were classified in ICE Quartile 1 and 17.8% were in Quartile 3. The participant-level clustering approach yielded more consistent ranges of scores for the NDI and ICE, and exhibited stronger associations with late-stage breast cancer for the ICE. Nevertheless, even if a clustering approach is used, grouping continuous data results in information loss and can impede comparisons between studies.

One approach that may offer a balance of interpretability and validity is latent class modeling, which allows for direct modeling of categorical classifiers. In latent class analysis (LCA), unmeasured or “latent” subgroups are identified based on observed shared characteristics [37]. An advantage of LCA is that categories are determined using model-based classifications, rather than arbitrary cut-points along a continuous scale. Further, this approach has been previously validated in cancer research, and one prior study found that latent class measures performed better than a single composite index at identifying residence in the most deprived neighborhoods among women with breast cancer [38]. Interpretation (including naming) of latent class categories requires additional discretion from the investigators compared to continuous or ordinal indices that have a natural order, which can make comparisons between indices challenging.

A limitation of this work is that all measures were defined at the census tract level, which are county subdivisions with populations typically in the range of 1200 to 8000 people [39]. Neighborhood indices are also available at a more granular census block group level (tract subdivisions in the range of 600 and 3000 people), which better capture notions of neighborhoods [12, 40]. However, with fewer people at the block group level, there is lower precision of American Community Survey estimates and thus inconsistent reliability of composite indices across different areas [41]. Also, we note that our study evaluated late-stage breast cancer in a North Carolina population, and given heterogeneity across the USA, variation is expected when applied to other populations. Additionally, the associations presented in the main analysis were not adjusted for individual SES (as our goal was to better understand the predictive value of neighborhood attributes), meaning these associations should not be interpreted as causal effects of neighborhood exposures. Finally, neighborhoods were classified based on residence at diagnosis and do not capture effects of earlier-life neighborhood conditions or health culture on late-stage breast cancer.

Multidimensional neighborhood indices have become a mainstay in health policy efforts to identify vulnerable populations. Understanding the differences between available indices is critical for accurately reporting health disparities and subsequently informing interventions. Overall, our results suggest the use of neighborhood indices can be improved with careful consideration of classification methods (e.g., reporting quantile and cluster groups) and complemented with indices that capture additional neighborhood features—especially access to healthcare. Ultimately, index selection should be guided by the themes and variables within the index, and their interpretation should be anchored to those variables, in context of population group, outcome, and geography.

## Supplementary Information

Below is the link to the electronic supplementary material.

Supplementary file1 (DOCX 329 kb)

## Data Availability

The data that support the findings of this study are available upon submission of a letter of intent and approval from the Carolina Breast Study Steering Committee (https://ciphr.unc.edu/cbcs-loi-form.php) and IRB approval. The data are not publicly available to protect the privacy of study participants.
