# Supplementary material for: Comparing Neighborhood Indices of Socioeconomic Status, Segregation, and Healthcare Access for Predicting Late-Stage Breast Cancer
Source: J Urban Health. 2026 Apr 10;103(2):395–405. doi: 10.1007/s11524-026-01072-4 (PMC13235670; doi:10.1007/s11524-026-01072-4)
Supplement: Supplementary file 1 — Supplementary file1 (DOCX 329 kb) [file 11524_2026_1072_MOESM1_ESM.docx]

**Supplemental Table 1. Themes and specific components of selected indices**

| Index | Theme | Components |
| --- | --- | --- |
| NDI | SES/Deprivation | -Median household income (dollars)  - Households receiving dividends, interest, or rental income  - Households receiving public assistance  -Median home value (dollars)  - Families with incomes below the poverty level  - High school degree or higher  - College degree or higher  - Management, business, science, or arts occupation  - Unemployed  - Households that are female headed with any children under 18  - Housing units that are owner occupied  - Households without a telephone  - Households without complete plumbing facilities |
| Yost | SES/Deprivation | - Education Index  - Households above 200% poverty line  - Persons with a blue collar job  - Persons employed  - Median rent  - Median value of owner-occupied housing unit  - Median household income |
| ICE | Segregation | -Black and white race  -Low and high income |
| Care Access | Healthcare access | Mammography utilization  Check-up utilization  Self-reported health  Health Insurance  Poverty  Broadband internet subscription  Distance from tract centroid (population weighted center) to nearest emergency department  Distance from tract centroid to nearest National Cancer Institute designated comprehensive cancer center  Number of oncologists - county-level  Number of primary care physicians – county-level |

**Supplemental Table 2. Included census-tract level measures and participant frequencies**

|  | Overall | Black | Non-Black |
| --- | --- | --- | --- |
| Index | N (%) | N (%) | N (%) |
| **NDI** |  |  |  |
| Quartile 1 | 972 (32%) | 308 (21%) | 664 (44%) |
| Quartile 2 | 617 (21%) | 264 (18%) | 353 (24%) |
| Quartile 3 | 660 (22%) | 356 (24%) | 304 (20%) |
| Quartile 4 | 748 (25%) | 567 (38%) | 181 (12%) |
| **Yost** |  |  |  |
| Quartile 4 | 728 (24%) | 231 (15%) | 497 (33%) |
| Quartile 3 | 742 (21%) | 222 (15%) | 400 (27%) |
| Quartile 2 | 622 (25%) | 377 (25%) | 365 (24%) |
| Quartile 1 | 905 (30%) | 665 (44%) | 240 (16%) |
| **ICE** |  |  |  |
| Quartile 1 | 943 (32%) | 222 (15%) | 625 (42%) |
| Quartile 2 | 673 (23%) | 200 (14%) | 334 (22%) |
| Quartile 3 | 534 (18%) | 337 (23%) | 336 (22%) |
| Quartile 4 | 847 (28%) | 736 (49%) | 207 (14%) |
| **Care Access** |  |  |  |
| High affordability,  high accessibility | 1031 (34%) | 415 (28%) | 616 (41%) |
| High affordability,  low accessibility, | 909 (30%) | 315 (21%) | 594 (40%) |
| Low affordability,  high accessibility, | 474 (16%) | 407 (27%) | 67 (5%) |
| Low affordability,  low accessibility | 584 (20%) | 358 (24%) | 226 (15%) |


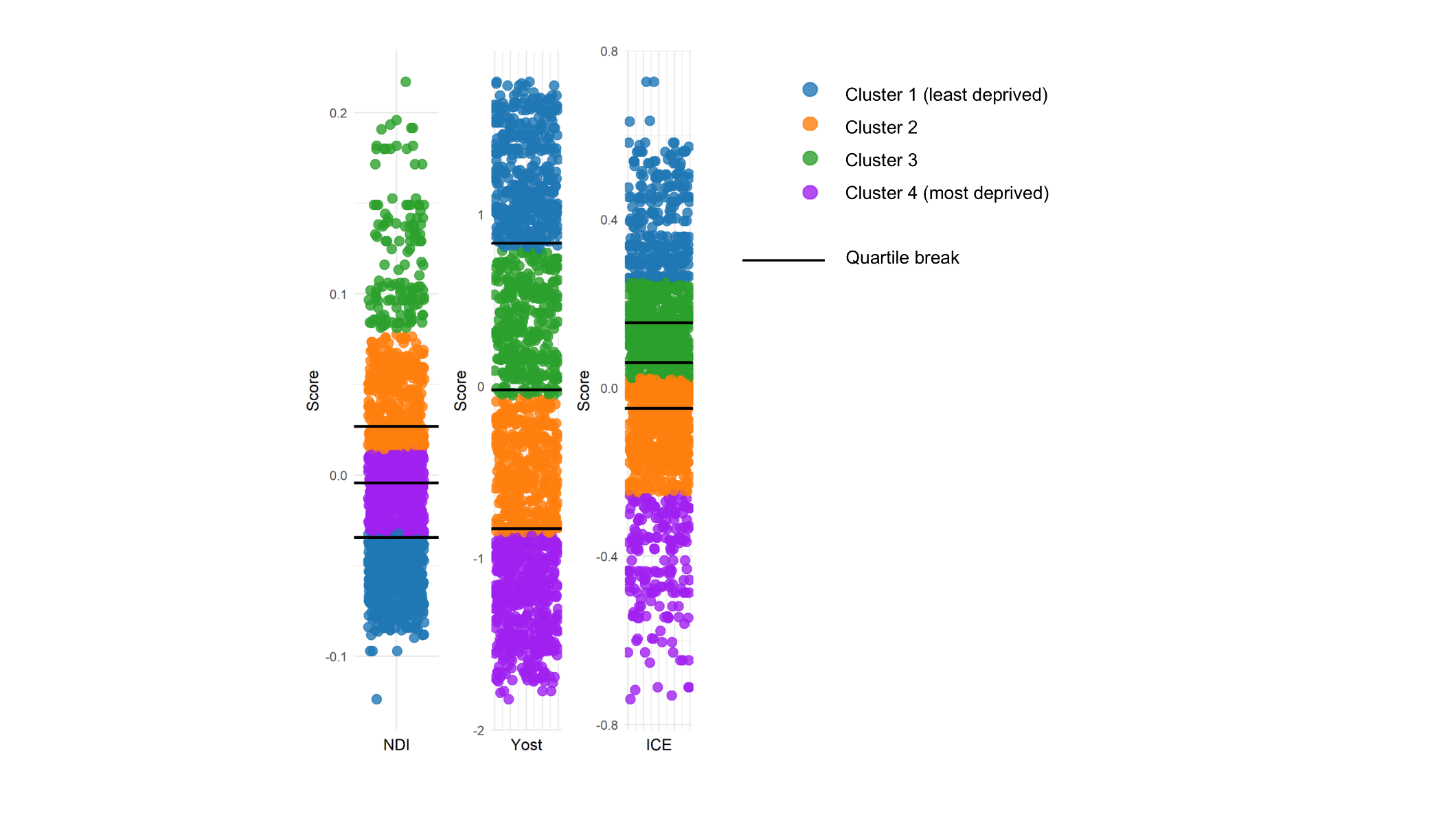


**Supplemental Figure 1. Categorization of CBCS participants by continuous measures using quartiles vs clustering.** Dot plots of NDI, Yost, and ICE scores for CBCS participants. For each measure, groupings were determined using national quartile cut points (indicated by horizontal lines) and k-means clustering (indicated by changing color) with 4 clusters.
